# S38. Many reasons for combining immunotherapy with chemotherapy to enhance anti tumour response

**DOI:** 10.1186/2051-1426-2-S2-I7

**Published:** 2014-03-12

**Authors:** A Dalgleish, D Fowler, A Gravett, R Allen, W Liu

**Affiliations:** 1 St. George's Hospital Medical School, Cellular and Molecular Medicine, London, UK

## Background

Chemotherapy and Immunotherapy were always considered incompatible in the treatment of cancer. However, analysis of sequential use has revealed that there may be significant additive potential if not synergistic, in enhancing the immune response to cancer.

## Material and methods

There are many reasons why chemotherapy may enhance immune therapy. These include1) the release of tumour antigens to a primed immune response, 2) the suppression and reduction of suppressor and regulatory T cells in the tumour and stromal tissues, 3) The priming of tumour cells to make them more visible to the immune response, 4) reduction of inflammatory and growth factor drive.

## Results

Enhancing the immune response prior to chemotherapy and releasing antigens as well as stimulating the immune response after Chemotherapy or radiotherapy are both effective models. Here we look at specific examples such as pre-stimulation with non specific mycobacterial vaccines that will enhance the responses to gemcitabine and zometa, the former boosting the Antigen specific responses and the later the gamma delta T cell activity.

Low dose chemotherapy such as with cyclophosphamide or pre vaccine treatment with the IMiDs such as Revlimid have been shown to enhance the immune response directly (co-stimlation) and indirectly by depressing T reg function. Both agents are in therapeutic vaccine trials for both HIV and Cancer. Another agent which is anti-inflammatory and immune stimulatory is low dose naltrexone (LDN) and has been noted to induce vitiligo in melanoma patient. This has lead to studies showing that in addition to modulating opiate receptors on immune cells it is a strong interactor with TLRs.

## Conclusions

There are numerous reasons how chemotherapy and immunotherapy can be additive if not synergistic. The sequential use of these agents may be more or as important as combining them, and the availability of the check point blockers has added another dimensions as well as the resurrection of cytokines such as low dose IL-2 for keeping the effector/memory cells active. There is a good case to prime with non specific vaccine activators, to then treat with chemotherapy followed by check point inhibitors and them low dose cytokines , such as IL-2 or other candidates such as IL-7,12,15, or 21.

